# Possible Involvement of Hepatitis B Virus Infection of Hepatocytes in the Attenuation of Apoptosis in Hepatic Stellate Cells

**DOI:** 10.1371/journal.pone.0146314

**Published:** 2016-01-05

**Authors:** Reina Sasaki, Tatsuo Kanda, Masato Nakamura, Shingo Nakamoto, Yuki Haga, Shuang Wu, Hiroshi Shirasawa, Osamu Yokosuka

**Affiliations:** 1 Department of Gastroenterology and Nephrology, Chiba University, Graduate School of Medicine, Chiba, 260–8670, Japan; 2 Department of Molecular Virology, Chiba University, Graduate School of Medicine, Chiba, 260–8670, Japan; SAINT LOUIS UNIVERSITY, UNITED STATES

## Abstract

**Background:**

The induction of apoptosis in hepatic stellate cells (HSCs) is a promising therapeutic strategy against hepatitis B virus (HBV)-related hepatic fibrosis. The underlying mechanisms of apoptosis in HSCs, however, are unknown under consideration of HBV infection. In this study, the effects of HBV on apoptosis and endoplasmic reticulum (ER) stress signaling in HSCs were examined.

**Methods:**

The effects of conditioned media (CM) from HepG2.2.15 on apoptosis induced by the proteasome inhibitor MG132 in LX-2 and HHSteC were studied in regard to c-Jun. In combination with c-Fos, c-Jun forms the AP-1 early response transcription factor, leading to AP-1 activation, signal transduction, endoplasmic reticulum (ER) stress and apoptosis.

**Results:**

In LX-2 cells, MG132 treatment was associated with the phosphorylation of c-Jun, activation of AP-1 and apoptosis. However, in the presence of CM from HepG2.2.15, these phenomena were attenuated. In HHSteC cells, similar results were observed. HBV genomic DNA is not involved in the process of HSC apoptosis. It is possible that HBeAg has an inhibitory effect on MG132-induced apoptosis in LX-2. We also observed the upregulation of several ER stress-associated genes, such as cAMP responsive element binding protein 3-like 3, inhibin-beta A and solute carrier family 17-member 2, in the presence of CM from HepG2.2.15, or CM from PXB cells infected with HBV.

**Conclusions:**

HBV inhibits the activation of c-Jun/AP-1 in HSCs, contributing to the attenuation of apoptosis and resulting in hepatic fibrosis. HBV also up-regulated several ER stress genes associated with cell growth and fibrosis. These mechanistic insights might shed new light on a treatment strategy for HBV-associated hepatic fibrosis.

## Introduction

Hepatitis B virus (HBV) infection is a major cause of chronic hepatitis and cirrhosis, and occasionally leads to hepatocellular carcinoma (HCC) [1]. HCC often occurs in patients with a background of HBV-related fibrotic liver. HBV infection is a serious health issue worldwide, and it is important to prevent patients infected with HBV from developing liver diseases with severe fibrosis. Higher levels of HBV DNA, HBV e antigen (HBeAg), and serum alanine aminotransferase, as well as liver cirrhosis, are strong risk predictors of HCC [2]. Long-term suppression of HBV DNA by nucleos(t)ide analogues could lead to a regression of hepatic fibrosis [3] as well as HCC [4–7].

An activated hepatic stellate cell (HSC) is one of the major sources of extracellular matrix in hepatic fibrosis and cirrhosis [8, 9]. The activation of HSCs is a key event in hepatic fibrogenesis [8]. On the other hand, resolution of hepatic fibrosis refers to pathways that either drive HSC to apoptosis, or contribute to reversion of HSC to a more quiescent phenotype, which is unknown in vivo [8]. However, previous studies supported the importance of apoptosis of HSCs during the regression of hepatic fibrosis [8, 10, 11]. HSCs are sensitive to CD95-L and tumor necrosis factor-related apoptosis-inducing ligand (TRAIL)-mediated apoptosis [12].

MG132, a proteasome inhibitor, could activate c-Jun N-terminal kinase (JNK), which initiates apoptosis and also inhibits NF-κB activation [13, 14]. MG132 blocks NF-κB activation and induces apoptosis in HSCs [15]. MG132 also leads to activator protein-1 (AP-1) activation and apoptosis in human epithelial cells [16, 17]. A previous study showed that JNK/AP-1 signaling pathways play a role in apoptosis in HSCs [18]. JNK was identified by its ability to specifically phosphorylate the transcription factor c-Jun on its N-terminal transactivation domain at serine residues [19]. c-Jun in combination with c-Fos forms the AP-1 early response transcription factor.

Here, we demonstrate that MG132 leads to AP-1 activation and apoptosis in human HSCs. We report that HBV inhibits the phosphorylation of c-Jun and the activation of AP-1, resulting in the attenuation of apoptosis in human HSCs. We found that HBV could play a role in the attenuation of apoptosis in human HSCs. We also determined that HBV up-regulates several ER stress genes associated with cell growth and fibrosis. These mechanistic insights might shed new light on the treatment strategy of HBV-associated hepatic fibrosis.

## Materials and Methods

### Cell cultures

Human hepatoma HepG2 and HepG2.2.15 cells [20] were grown in Roswell Park Memorial Institute medium (RPMI-1640) (Sigma-Aldrich, St. Louis, MO, USA) supplemented with 10% fetal bovine serum (FBS) at 5% CO_2_ and 37°C. HepG2.2.15 cells are derived from HepG2 cells and are characterized by stable 1.3-fold HBV (genotype D) genome expression and replication [20–22].

A spontaneously immortalized human hepatic stellate cell line, LX-2 [23], kindly provided by Prof. S. L. Friedman, was cultured in Dulbecco’s modified Eagle medium (DMEM) (Sigma-Aldrich) supplemented with 10% or 1% fetal bovine serum (FBS). Primary human hepatic stellate cells HHSteC, which were purchased from ScienCell Research Laboratories (Carlsbad, CA, USA), were maintained in Stellate Cell Medium (ScienCell Research Laboratories) with 2% FCS plus stellate cell growth supplement (ScienCell Research Laboratories).

Human hepatocyte PXB cells (PhoenixBio, Higashi-Hiroshima, Japan) [24] were derived from chimeric mice with hepatocyte-humanized livers (PXB-mouse). PXB cells were cultured without passage as described previously [24], and maintained in DMEM supplemented with 2% FBS, 20 mM HEPES, 44 mM NaHCO3, 15 μg/mL L-proline, 0.25 μg/mL insulin, 50 nM dexamethasone, 5 ng/mL EGF, 0.1 mM Asc-2P and 2% DMSO (2% DMSO-supplemented hepatocyte clonal growth medium (dHCGM)).

HepG2 cell lines that stably expressed the HBV core region with or without a precore region (HBeAg-negative HepG2 or HBeAg-positive HepG2, respectively) were previously described [25].

### Preparation of conditioned media and HBV infection

HepG2 and HepG2.2.15 cells were grown in T-25 or T-75 flasks in RPMI-1640 supplemented with 10% FBS at 37°C. At approximately 90% confluency, the cells were washed and incubated in RPMI-1640 supplemented with 10% FCS for 48 hours. Conditioned media were clarified by centrifugation at 5,000 g to remove cell debris, and stored at -20°C until use. HBV genotype C isolates were purchased from PhoenixBio [24]. Informed consent from the patient was obtained by Prof. Yasuhito Tanaka, Nagoya City University, Japan [24]. The infection methods were as previously reported [24]. Conditioned media were prepared similarly from PXB cells at 17 days after infection with or without HBV [24]. Ultraviolet (UV)-inactivated virus was prepared by irradiating conditioned media samples with a UV trans-illuminator as previously described [26].

### Reporter assay for AP-1 activation

LX-2 cells were seeded onto a 6-well plate and 24 hours later co-transfected with 0.1 μg reporter plasmid pAP-1-luc (PathDetect Cis-Reporting Systems; Agilent Technologies, Santa Clara, CA, USA) and 0.1 μg pCXN2-HBx or pCXN2 control vector (kindly provided by Prof. J. Miyazaki) using Effectene transfection reagents (Qiagen, Hilden, Germany) [14]. Forty-eight hours after transfection, the cells were harvested using reporter lysis buffer (Toyo Ink, Tokyo, Japan) and luciferase activities were determined with a luminometer (Luminescencer-JNR II AB-2300, ATTO, Tokyo, Japan).

### RNA purification, cDNA synthesis and human ER stress-associated signaling target PCR array

LX-2 cells were incubated with conditioned media from HepG2, HepG2.2.15, or PXB infected with or without HBV for 24 hours. Cellular RNA was extracted using the RNeasy Mini Kit (Qiagen) according to the manufacturer’s instructions. One microgram of RNA was reverse-transcribed with a PrimeScript RT^2^ First Strand Kit (Qiagen). A human ER stress-associated signaling target PCR array was performed according to the manufacturer's protocol [14]. The data were analyzed using PCR Array Data Analysis Software (http://www.sabiosciences.com/pcrarraydataanalysis.php).

### Western blotting

Cells were collected in sodium dodecyl sulfate sample buffer. After sonication, cell lysates were subjected to electrophoresis on 5–20% polyacrylamide gels and transferred onto polyvinylidene difluoride membranes (ATTO). The membranes were probed with specific antibodies for poly(adenosine diphosphate-ribose) polymerase (PARP), JNK, c-Jun, phosphorylated-c-Jun (Ser63) (Cell Signaling Technology, Danvers, MA, USA) and GAPDH (Santa Cruz Biotechnology, Santa Cruz, CA, USA). After washing, the membranes were incubated with secondary horse-radish peroxidase-conjugated antibodies. Signals were detected by means of enhanced chemiluminescence (GE Healthcare Japan, Tokyo, Japan) and scanned with an image analyzer LAS-4000 and Image Gauge (version 3.1) (Fuji Film, Tokyo, Japan). Band intensities were determined using ImageJ software [14].

### Immunofluorescence study

The cells were washed and fixed with 3.7% formaldehyde, followed by blocking with 3% horse serum albumin. The cells were incubated with an Annexin-V mouse antibody (Santa Cruz) and γ-H_2_A.X (phospho S139) rabbit antibody for 16 hours at 4°C. The cells were washed and incubated with anti-rabbit immunoglobulin secondary antibody conjugated with anti-mouse Alexa Fluor 488 antibody and anti-rabbit Alexa Fluor 555 antibody (Cell Signaling) for 1 hour at room temperature. Nuclear staining was performed with Hoechst 33342, trihydrochloride, trihydrate (Molecular Probes, Eugene, OR, USA). Finally, the cells were washed and mounted for confocal microscopy (ECLIPSE TE 2000-U, Nikon, Tokyo, Japan), and the images were superimposed digitally to allow for fine comparisons [14].

### Knockdown of c-Jun

The siRNA against c-Jun (si-c-Jun1 and si-c-Jun2) and control siRNA (si-C) were obtained from Santa Cruz Biotechnology. Transfections were performed with 50 nM si-c-Jun1, 50 nM si-c-Jun2, or 50 nM si-C using Effectene Transfection Reagents (Qiagen) according to the manufacturer’s protocol.

### Overexpression of MEKK

To overexpress c-Jun, MEKK upstream of c-Jun was overexpressed. LX-2 cells were transfected with or without 0.01 μg pMEKK (Agilent Technologies) using Effectene Transfection Reagents (Qiagen) according to the manufacturer’s protocol.

### Measurement of caspase-3/-7 activities

The Caspase-Glo 3/7 assay (Promega, Madison, WI, USA) was used to determine caspase-3 and -7 activities according to the manufacturer’s instructions [14]. Briefly, the Caspase-Glo 3/7 reagent was added at a 1:1 ratio to the conditioned media from a cell culture and left for 1 hour at room temperature. Luminescence was recorded as a function of caspase-3 and -7 activities using Luminescencer-JNR II AB-2300 (ATTO). Blank wells were used as nonspecific background. The ratios of caspase-3 and -7 activities from each group relative to untreated control groups, defined as 1, were determined by luminescence.

### Apoptosis assay

LX-2 cells were treated with 20 μM MG132 (Sigma-Aldrich) for 24 hours, and apoptosis was evaluated using the APOPercentage Apoptosis Assay (Biocolor, Belfast, Northern Ireland) according to the manufacturer’s instructions. The transfer and exposure of phosphatidylserine to the exterior surface of the membrane has been linked to the onset of apoptosis. Phosphatidylserine transmembrane movement results in the uptake of APOPercentage dye by apoptotic cells. Purple-red stained cells were identified as apoptotic cells by light microscopy. The number of apoptotic cells was counted as previously described [14].

### Statistical analysis

The results were expressed as mean ± standard deviation (SD). Statistical analyses were performed by Student’s t-test with DA Stats software (O. Nagata, Nifty Serve: PAF01644). A P-value of < 0.05 was considered statistically significant.

## Results

### Conditioned media from HepG2.2.15 decreases AP-1 activation in LX-2 cells

**Fig 1:**
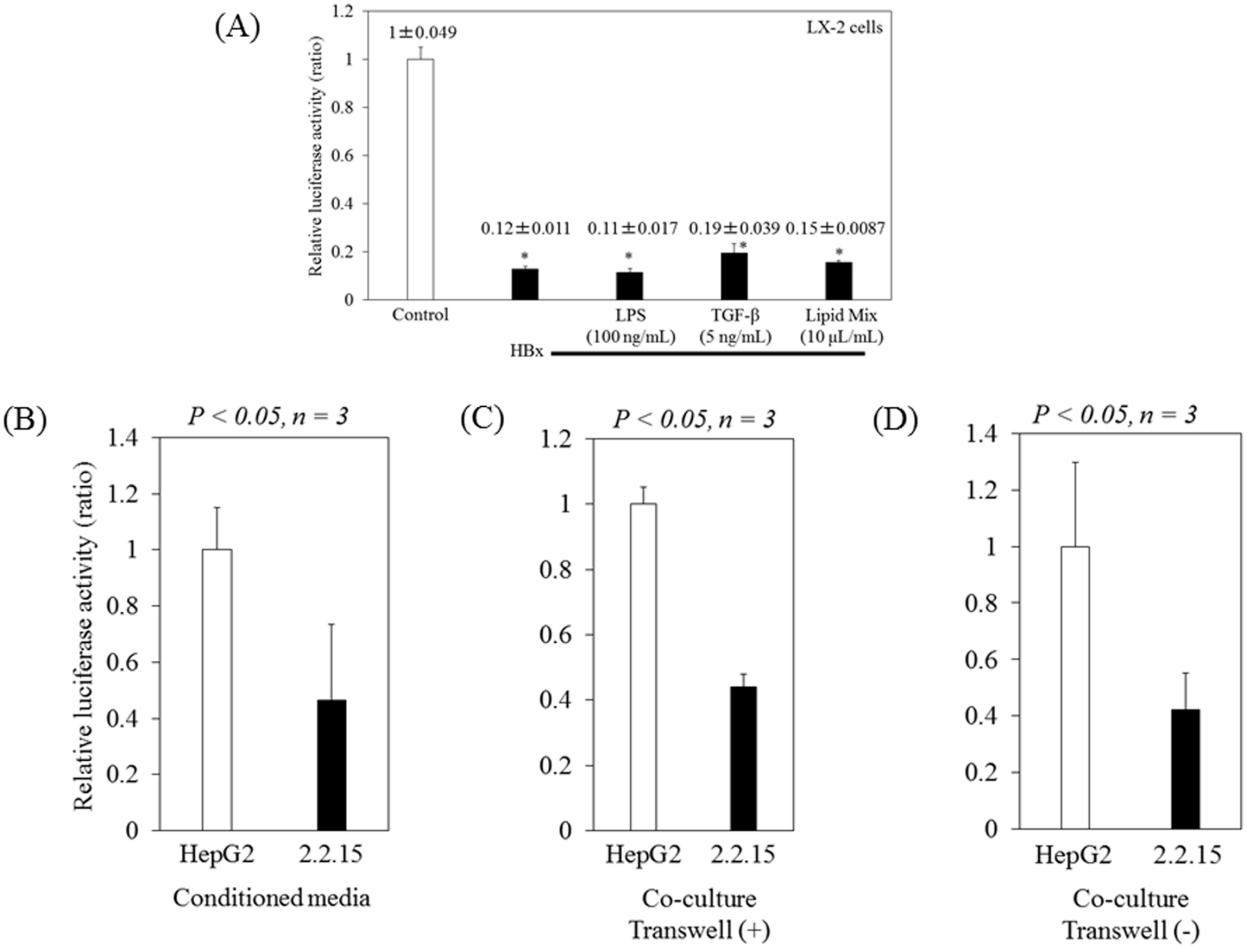
Hepatitis B virus (HBV) inhibits AP-1 activation in LX-2 cells. (A) LX-2 cells were transfected with pAP-1-luc with pCXN2-HBx or pCXN2. After 24 hours of transfection, LX-2 cells were treated with or without 100 ng/mL LPS (Novus Biologicals, Littleton, CO, USA), 0.1% lipid mixture 1 (Sigma), or 5 ng/mL TGF-β (Wako Pure Chemical, Osaka, Japan). (B) After 24 hours of transfection with pAP-1-luc, the cells were treated with conditioned media from HepG2 or from HepG2.2.15 (indicated as 2.2.15). (C), (D) After 24 hours of transfection with pAP-1-luc, the cells were co-cultured with HepG2 or HepG2.2.15 with (C) or without the transwell system (D). After 48 hours of transfection, AP-1 activity was measured by luciferase assay. Data are expressed as mean ± standard deviations of triplicate determinations. *P < 0.01 (vs. control).

We have previously shown that HBx enhances AP-1 activation in the human hepatoma cell line Huh7 [27]. However, it is not clear whether HBx enhances AP-1 activation in human HSC LX-2 cells. To examine the effects of HBx protein on the AP-1 signaling pathway in LX-2 cells, we investigated AP-1-mediated transcriptional activation. After 48 hours of transfection of pAP-1-luc with pCXN2-HBx or pCXN2 control vector, we measured AP-1 activity by luciferase assay. We observed that HBx down-regulated AP-1 activation at 0.1- to 0.2-fold of the levels of control in LX-2 cells (Fig 1A), although we also added lipopolysaccharide (LPS) and transforming growth factor beta (TGF-β), which are associated with hepatic fibrosis [28]. HBx functions as a transactivator protein and plays a role in HBV-related hepatocarcinogenesis [27]; however, it is not known whether HBV could infect HSCs in vivo.

We next examined the effect of conditioned media from HepG2.2.15, which included an infectious HBV [21, 22], on AP-1 activation in LX-2 cells (Fig 1B). Similar to the results described above, we observed that conditioned media from HepG2.2.15 down-regulated AP-1 activation at 0.47-fold of the levels after treatment with conditioned media from HepG2 (1±0.15 vs. 0.47±0.26; P<0.05, n = 3) (Fig 1B). We also investigated the effects of co-culture of LX-2 cells with HepG2.2.15 cells on AP-1 activation with or without transwell systems (Fig 1C and 1D, respectively). Co-culture of LX-2 cells with HepG2.2.15 cells down-regulated AP-1 activation at 0.42- to 0.44-fold of the levels of co-culture with HepG2 control cells (1±0.051 vs. 0.44±0.040; P<0.05, n = 3 (Fig 1C) and 1±0.30 vs. 0.42±0.13; P<0.05, n = 3 (Fig 1D)). Thus, our results suggested that HBV might decrease AP-1 activation in LX-2 cells.

### Conditioned media from HepG2.2.15 inhibits phosphorylated-c-Jun in LX-2 cells

**Fig 2:**
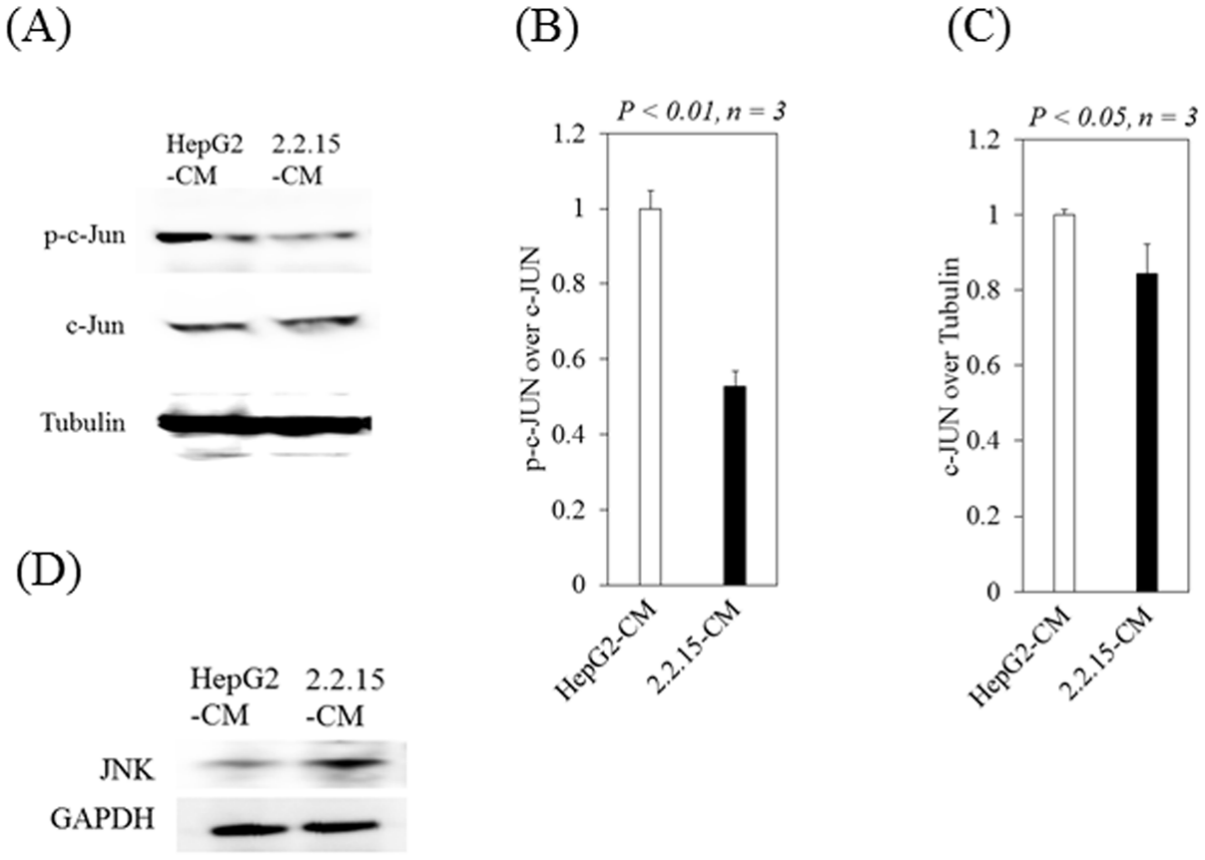
Conditioned media from HepG2.2.15 (2.2.15-CM) inhibits phosphorylated-c-Jun (p-c-Jun) in LX-2 cells. (A)-(C) Western blot analyses of phosphorylated-c-Jun, c-Jun and GAPDH expression in LX-2 cells treated with conditioned media from HepG2 (HepG2-CM) or 2.2.15-CM. (B), (C) Densitometric analyses were performed by ImageJ software. (D) Western blot analyses of JNK and GAPDH expression in LX-2 cells treated with HepG2-CM or 2.2.15-CM. Data are expressed as mean ± standard deviations of triplicate determinations.

Next, we examined the role of HBV in phosphorylated-c-Jun or c-Jun protein expression in LX-2 cells, treated with conditioned media from HepG2.2.15 or from HepG2 control. Treatment with conditioned media from HepG2.2.15 was associated with a 47.3% reduction in phosphorylated-c-Jun expression (Fig 2A and 2B) and a 15.6% reduction in c-Jun expression (Fig 2A–2C). However, treatment with conditioned media from HepG2.2.15 was associated with an approximately 40% increase in JNK protein expression (Fig 2D). Our results indicated that the phosphorylation of c-Jun was significantly inhibited in LX-2 cells treated with conditioned media from HepG2.2.15 compared to LX-2 cells treated with conditioned media from HepG2. The observation that conditioned media from HepG2.2.15 significantly suppressed AP-1 activation (Fig 1B–1D) indicates that the conditioned media from HepG2.2.15 may play an inhibitory role in the phosphorylation of c-Jun in LX-2 cells.

### Conditioned media from HepG2.2.15 attenuates MG132-induced apoptosis in HSCs

**Fig 3:**
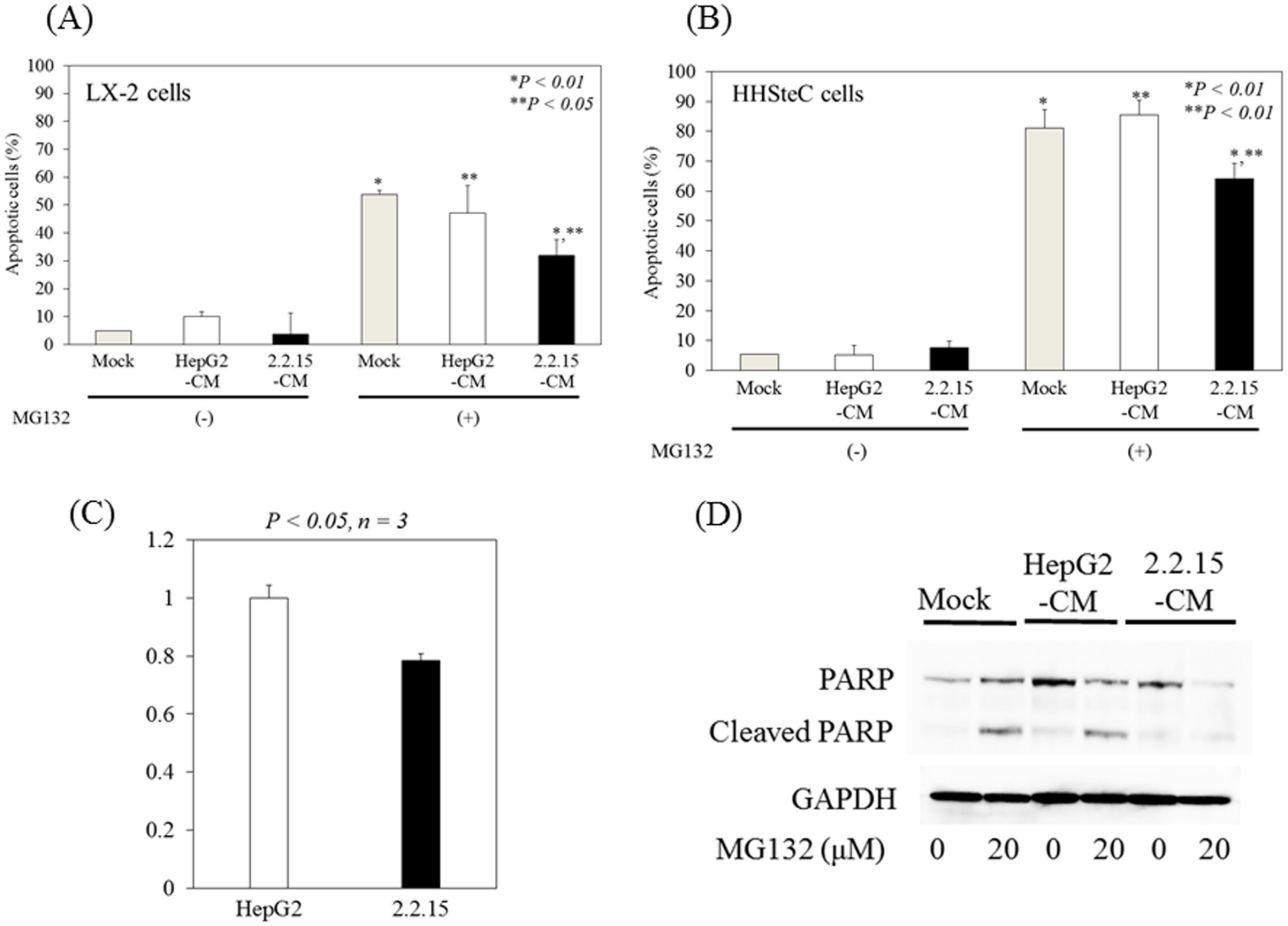
Conditioned media from HepG2.2.15 (2.2.15-CM) protects hepatic stellate cells from MG132-induced apoptosis. (A) LX-2 cells were cultured with mock control, conditioned media from HepG2 (HepG2-CM), or conditioned media from HepG2.2.15 for 24 hours with or without MG132 (20 μM). *P < 0.01, **P < 0.05. (B) HHSteC cells were cultured with mock control, conditioned media from HepG2 (HepG2-CM), or conditioned media from HepG2.2.15 for 24 hours with or without MG132 (20 μM). *P < 0.01, **P < 0.01. Apoptosis was quantified using the APOPercentage Apoptosis Assay. Data are expressed as mean ± standard deviations of triplicate determinations. (C) Caspase-3/-7 activities following 20 μM of MG132 treatment were measured in conditioned media from LX-2 cells incubated with conditioned media from HepG2 or HepG2.2.15 for 24 hours. Caspase-3/-7 activities were determined using the Caspase-Glo 3/7 assay (Promega, Madison, WI, USA). (D) Western blot analysis of PARP and GAPDH expression in LX-2 cells treated with conditioned media from HepG2 (HepG2-CM) or 2.2.15-CM after 24 hours of MG132 treatment.

A previous study showed that MG132 treatment activates c-Jun and sensitizes prostate cancer cells to apoptosis caused by anticancer agents [17]. Proteasome inhibition by MG132 also causes HSC apoptosis [15]. We examined whether conditioned media from HepG2.2.15 had any effect on MG132-induced apoptosis in HSC LX-2 cells. LX-2 cells treated with or without conditioned media from HepG2.2.15 or conditioned media from HepG2 were used to determine MG132-mediated apoptotic cell death. The apoptosis assay revealed a 40.6% or 32.2% reduction in apoptosis in MG132-treated LX-2 cells incubated with conditioned media from HepG2.2.15 compared to conditioned media from HepG2 or mock control, respectively (Fig 3A). We also examined whether conditioned media from HepG2.2.15 had any effects on MG132-induced apoptosis in primary human HSC HHSteC cells. The apoptosis assay revealed a 20.8% or 25.0% reduction in apoptosis in MG132-treated HHSteC cells incubated with conditioned media from HepG2.2.15 compared to conditioned media from HepG2 or mock control, respectively (Fig 3B). These results suggested that MG132-induced apoptosis is inhibited by conditioned media from HepG2.2.15 in HSCs.

To determine whether conditioned media from HepG2.2.15 interferes with the MG132-induced apoptotic signaling pathway, conditioned media from LX-2 cells was analyzed for caspase-3/-7 activation by homogenous luminescent assay. Following MG132 treatment, activation of caspase-3/-7 was observed with LX-2 cells incubated with conditioned media from HepG2, compared to LX-2 cells incubated with conditioned media from HepG2.2.15 (0.78±0.024 vs. 1±0.043; P<0.05, n = 3) (Fig 3C).

We also determined the integrity of the death substrate PARP in LX2 cells incubated with or without conditioned media from HepG2.2.15 or conditioned media from HepG2, treated with or without MG132. We observed cleavage of the naïve approximately 116 kDa PARP to an approximately 85 kDa proteolytic fragment after 24 hours of MG132 treatment in LX-2 cells treated with conditioned media from HepG2 or mock control (Fig 3D). However, LX-2 cells treated with conditioned media from HepG2.2.15 did not show PARP cleavage. Thus, the results suggested that activation of caspase-3 and the cleavage of the DNA repair enzyme PARP were inhibited in LX-2 cells upon MG132 exposure in the presence of conditioned media from HepG2.2.15.

### c-Jun is important for apoptotic HSC death induced by MG132

**Fig 4:**
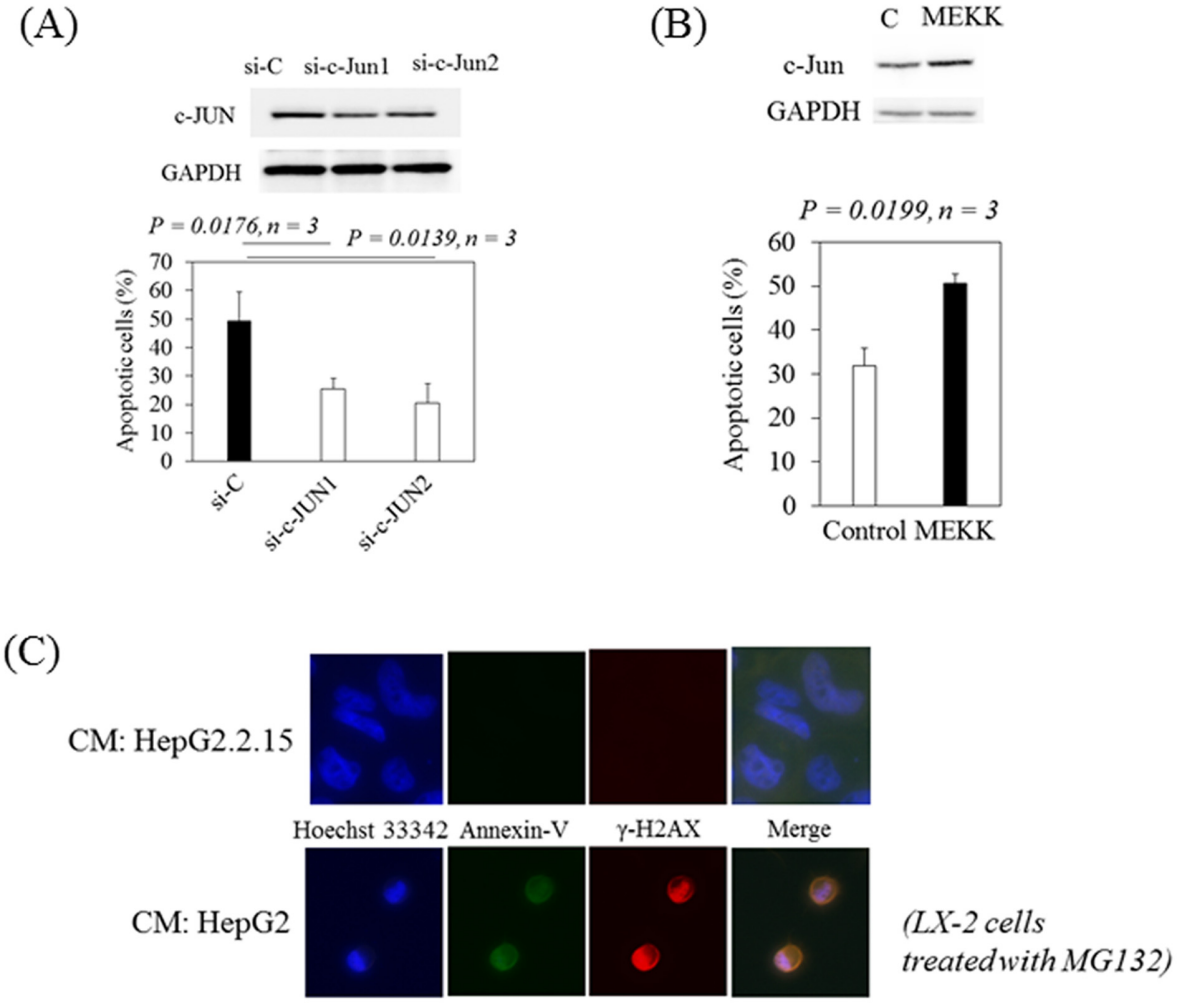
c-Jun is important for apoptotic hepatic stellate cell death induced by MG132. (A) Apoptotic cell deaths were lower in LX-2 cells transfected with siRNAs against c-Jun (si-c-Jun1 and si-c-Jun2) compared with LX-2 cells transfected with siRNA-control (si-C) after incubation with conditioned media from HepG2 in the presence of MG132 (lower panel). Western blot analysis of c-Jun and GAPDH expression in LX-2 cells treated with conditioned media from HepG2 after 24 hours of MG132 treatment (upper panel). (B) Overexpression of c-Jun by the transfection of pMEKK into LX-2 cells enhanced apoptosis in LX-2 cells treated with conditioned media from HepG2.2.15 in the presence of MG132 (lower panel). Western blot analyses of c-Jun and GAPDH expression in LX-2 cells treated with conditioned media from HepG2.2.15 after 24 hours of MG132 treatment (upper panel). Apoptosis was quantified using the APOPercentage Apoptosis Assay. Data are expressed as mean ± standard deviations of triplicate determinations. (C) Conditioned media from HepG2.2.15 (2.2.15-CM) protects hepatic stellate cells from MG132-induced apoptosis and DNA damage. Confocal microscopic findings with a high-power view (x200) of the expression of phosphorylated histone H2AX (γ-H2AX) (red), a DNA damage marker, and Annexin V (green), an apoptosis marker, in LX-2 cells treated with conditioned media from HepG2.2.15 (upper panel) or HepG2 (lower panel) in the presence of MG132.

We analyzed apoptotic cell death in LX-2 cells treated with conditioned media from HepG2 in the presence of MG132. Apoptotic cell deaths were reduced in LX-2 cells transfected with si-c-Jun1 or si-c-Jun2 compared with LX-2 cells transfected with si-C (P<0.05, n = 3) in the presence of MG132 (Fig 4A; 49.4%, 25.5% or 20.6% apoptosis in LX-2 cells transfected with si-C, si-c-Jun1 or si-c-Jun2, respectively). We also examined whether overexpression of c-Jun by the transfection of pMEKK into LX-2 cells had an enhanced effect on apoptosis in LX-2 cells treated with conditioned media from HepG2.2.15 in the presence of MG132. We observed that overexpression of c-Jun increased apoptosis in LX-2 cells in the presence of MG132 (P<0.05, n = 3) (Fig 4B). These results suggest that c-Jun plays a role in HSCs apoptosis induced by MG132 and that conditioned media from HepG2.2.15 could inhibit the AP-1 signaling pathway.

We also examined the expression of phosphorylated histone H2AX (γ-H2AX), which marks the site of DNA double-strand breaks and evokes the DNA repair system [29], and Annexin V, which binds to phosphatidylserine and marks apoptotic cells by immunofluorescence in LX-2 cells treated with conditioned media from HepG2.2.15 or HepG2 in the presence of MG132 (Fig 4C). In LX-2 cells treated with conditioned media from HepG2, γ-H2AX and Annexin V were merged and predominantly observed, compared with LX-2 cells treated with conditioned media from HepG2.2.15 (Fig 4C).

### ER stress-related genes up-regulated by conditioned media associated with HBV

**Fig 5:**
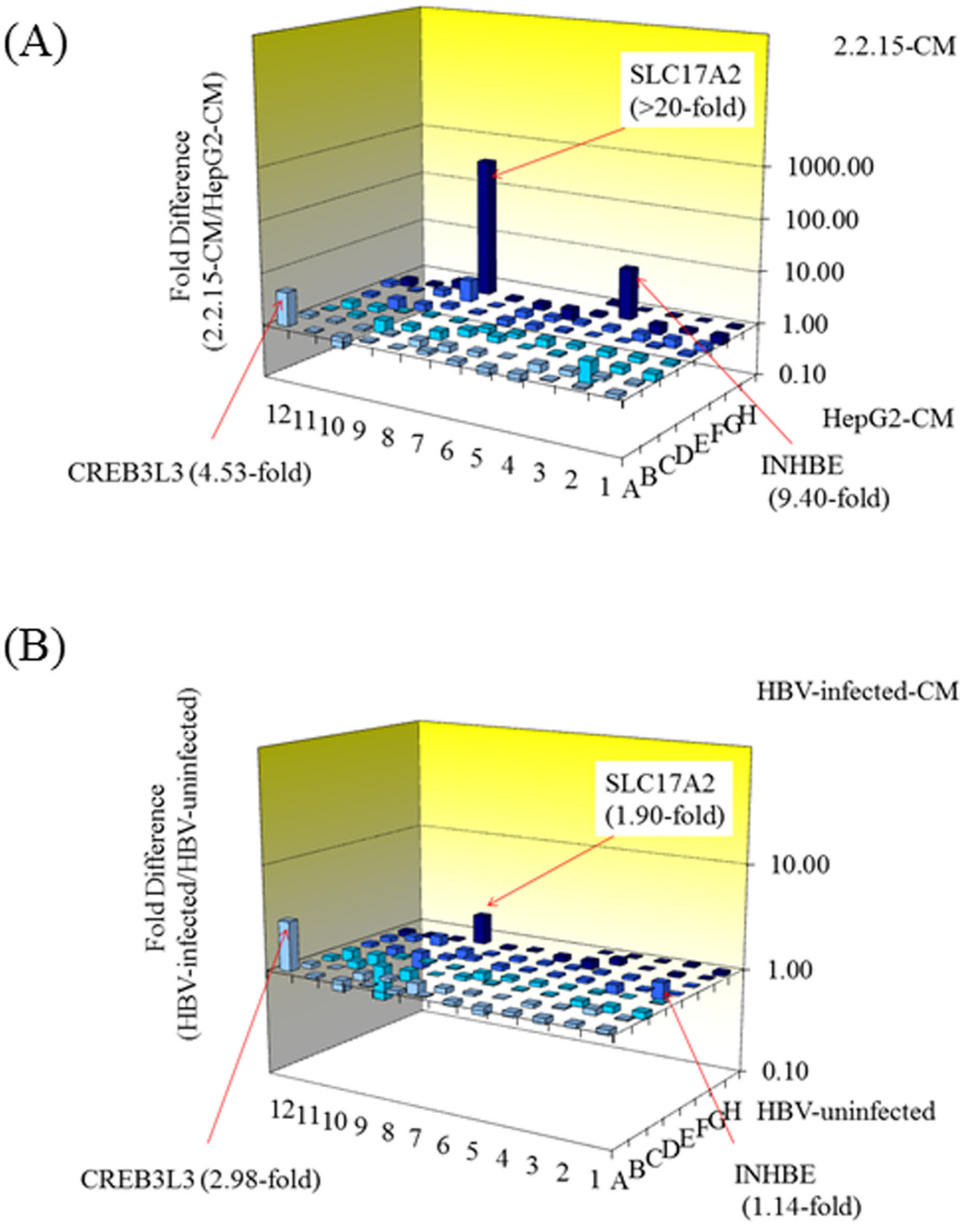
Real-time PCR array analysis for ER stress-associated genes in conditioned media associated with or without HBV. (A) Conditioned media from HepG2.2.15 (2.2.15-CM) and conditioned media from HepG2 (HepG2-CM) were used for the treatment of LX-2 cells for 48 hours. (B) Conditioned media from HBV-infected PXB (HBV-infected) and conditioned media from HBV-uninfected PXB (HBV-uninfected) were used for the treatment of LX-2 cells for 48 hours. Eighty-four ER-stress-associated gene expression profiles using real-time PCR-based focused microarrays (see Materials and Methods section). Three genes are indicated by arrows: solute carrier family 17, member 2 (SLC17A2); inhibin, beta E (INHBE); and cAMP responsive element binding protein 3-like 3 (CREB3L3), are indicated by arrows.

To investigate the effect of conditioned media associated with or without HBV on LX-2 cells, conditioned media from HepG2.2.15 and conditioned media from HepG2 were used to treat LX-2 cells for 48 hours (Fig 5A). We have examined ER-stress-associated gene expression profiles using real-time PCR-based focused microarrays. A comparison of ER stress-related genes in LX-2 cells treated with conditioned media from HepG2.2.15 and conditioned media from HepG2 at 48 hours is shown in Fig 5A. Of 84 genes examined, 3 genes (solute carrier family 17, member 2 (SLC17A2); inhibin, beta E (INHBE); cAMP responsive element binding protein 3-like 3 (CREB3L3)) were up-regulated 4.0-fold or greater in LX-2 cells treated with conditioned media from HepG2.2.15 (Fig 5A & Table 1). These 3 genes were also up-regulated in LX-2 cells treated with conditioned media from HBV-infected PXB cells, compared with LX-2 cells treated with conditioned media from HBV-uninfected PXB cells (Fig 5B & Table 1). SLC17A2 may be involved in actively transporting phosphate into cells via Na(+) cotransport [30]. INHBE is associated with the TGF-β signaling pathway [31]. CREB3L3 is a transcription factor that may act during endoplasmic reticulum stress by activating unfolded protein response target genes, and it promotes lipid droplet growth and hepatic steatosis [32]. CREB3L3 protein, a member of the CREB3 family of transcription factors, has a bZIP domain very similar to that of CREB3L2. The translational activity was achieved through the GRP78 promoter, box-B element, ATF6 and CRE binding sites, as well as the box-B element [33].

**Table 1:** Up-regulated genes (> 4-fold differences) in LX-2 cells treated with conditioned media from HepG2.2.15 compared with in LX-2 cells treated with that from HepG2.

Gene name	Protein	Function	Localization	Up-regulated genes	
				HepG2.2.15 vs. HepG2	HBV-infected vs. Uninfected PXB
CREB3L3		Transcription factor that may act during endoplasmic reticulum stress by activating unfolded protein response target genes.	Nucleus and endoplasmic reticulum	4.53-fold	2.98-fold
INHBE	Inhibin, beta	TGF-beta signaling pathway	Extracellular space	9.40-fold	1.14-fold
SLC17A2	NPT3	May be involved in actively transporting phosphate into cells via Na(+) cotransport	Plasma membrane	>20-fold	1.90-fold

SLC17A2, solute carrier family 17, member 2; INHBE, inhibin, beta E; CREB3L3, cAMP responsive element binding protein 3-like 3.

### Possible involvement of HBeAg in the attenuation of HSC apoptosis induced by MG132

Although it is likely that HSCs did not express solute carrier family 10 (sodium/bile acid cotransporter), member 1 (SLC10A1/NTCP) [34], we examined whether UV-inactivated HBV has an inhibitory effect on apoptosis in HSCs. We compared the inhibitory effects of conditioned media from HepG2.2.15 with or without UV-inactivation on MG132-induced apoptosis of LX-2 cells. We observed no statistically significant difference in apoptosis in the presence of MG132 (21.6% vs. 20.7%; n = 3). These results suggest that HBV genomic DNA is not involved in the process of HSC apoptosis.

**Fig 6:**
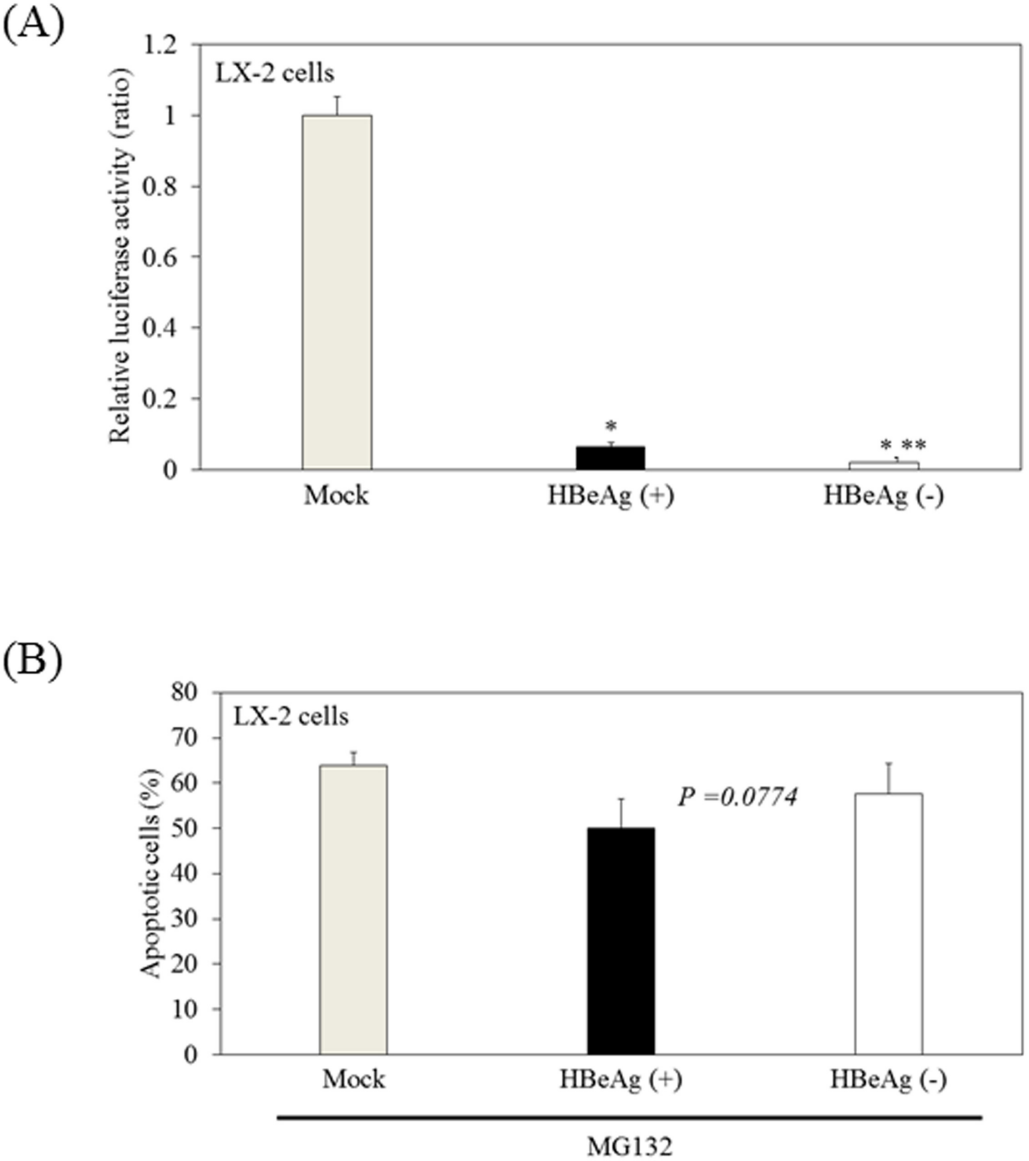
Possible involvement of hepatitis B virus e antigen (HBeAg) in the attenuation of apoptosis in hepatic stellate cells induced by MG132. (A) HBeAg inhibits AP-1 activation in LX-2 cells. LX-2 cells were transfected with pAP-1-luc with or without pCXN2-HBeAg(+) or pCXN2-HBeAg(-) [25]. After 48 hours of transfection, AP-1 activity was measured by luciferase assay. Data are expressed as mean ± standard deviations of triplicate determinations. *, **P < 0.01 (vs. mock control). (B) Inhibitory effects of conditioned media from HBeAg-positive HepG2 on MG132-induced apoptosis of LX-2. Apoptosis was quantified using the APOPercentage Apoptosis Assay. Data are expressed as mean ± standard deviations of triplicate determinations.

Because we previously reported that HBeAg could impair both innate and adaptive immune responses to promote chronic HBV infection, we focused on HBeAg [25, 35]. To examine the effects of HBeAg on the AP-1 signaling pathway in LX-2 cells, we investigated AP-1-mediated transcriptional activation. After 48 hours of transfection of pAP-1-luc with pCXN2-HBeAg(+), pCXN2-HBeAg(-) or mock [25], we measured AP-1 activity using a luciferase assay. pCXN2-HBeAg(+) could express both HBeAg and HBV core protein, but pCXN2-HBeAg(-) could express only HBV core protein. We observed that transfection with pCXN2-HBeAg(+) or pCXN2-HBeAg(-), respectively, down-regulated AP-1 activation at 0.065-fold or 0.019-fold of the levels of control in LX-2 cells (Fig 6A).

Next, we compared the inhibitory effects of conditioned media from HBeAg-negative HepG2 or from HBeAg-positive HepG2 on MG132-induced apoptosis in LX-2 cells. We observed that HBeAg-positive HepG2 conditioned media tended to have an inhibitory effect on MG132-induced apoptosis in LX-2 compared to HBeAg-negative HepG2 or mock control (Fig 6B). These results raised the possibility that HBeAg has an inhibitory effect on MG132-induced apoptosis in LX-2 cells, although the mechanism is still under investigation.

## Discussion

The pathophysiology between HSCs and HBV has been studied for many years. Conditioned media from HepG2.2.15, which includes an infectious HBV and soluble mediator secreted from HepG2.2.15, attenuates MG132-induced apoptosis in HSCs. Our observations suggested the involvement of AP-1 signaling in the inhibition of MG132-induced apoptosis by HBV in LX-2 cells through the suppression of phosphorylated-c-Jun expression.

Igaki et al. [36] reported that apoptosis was observed in the livers from 5 HBeAg-negative patients with fulminant hepatitis B. Shi et al. [37] also reported that the reduction of HBeAg in conditioned media from HepG2.2.15 cells induced by interferon-γ plus tumor necrosis factor-α resulted in apoptosis in HepG2.2.15 cells. Both HBV and HBeAg levels drove the programmed death 1 (PD-1) expression and T-cell impairment in chronic hepatitis B, and the suppression of HBV replication induced by treatment reduced PD-1 expression [38]. Thus, HBeAg could be associated with apoptosis in hepatocytes.

Our previous studies [25, 35] demonstrated that HBeAg down-regulates the production of cytokines such as interleukin (IL)6 in HepG2. Similarly, conditioned media from HepG2.2.15 cells also includes soluble mediators other than HBeAg. In a previous PCR array study ([35] and unpublished data), we found only gene MyD88, which was up-regulated ≥ 2-fold in both sets of HepG2.2.15 cells vs. HepG2 cells and HBeAg-positive HepG2 cells vs. HBeAg-negative HepG2 cells. Similarly, we found 6 genes (TLR1, IFNB1, TNF, IL6, TLR7, IL8 and CD180) that were down-regulated ≤ 2-fold in both sets of HepG2.2.15 cells vs. HepG2 cells and HBeAg-positive HepG2 cells vs. HBeAg-negative HepG2 cells [35]. Further studies may be needed to elucidate this issue.

HBV promotes the proliferation of HSCs through the platelet-derived growth factor-B (PDGF-B)/PDGF receptor-β (PDGFR-β) signaling pathway [39]. HBV directly promotes collagen type I expression in LX-2 cells [40]. The HBV S antigen (HBsAg) also directly affects the proliferation of HSCs and promotes collagen type I expression in LX-2 cells [41,42]. It was also reported that HBeAg directly activated HSCs [43]. Our results may support those studies. We also performed additional experiments with the use of purified HBeAg and purified HBsAg, but we did not observe any suppression of MG-132-induced apoptosis in LX-2 cells. It is possible that purified HBeAg and purified HBsAg may not have a similar potential to that of unpurified HBeAg and unpurified HBsAg, respectively. Further studies will be needed.

HBeAg could impair innate as well as adaptive immune responses to promote chronic HBV infection [25,35]. The persistence of serum HBeAg and high level of serum HBV DNA are thought to reflect a high HBV replication status in hepatocytes, causing cirrhosis and HCC [44], although this may be limited to HBeAg-positive hepatitis B patients. HBeAg may inhibit HSC apoptosis and promote hepatic fibrosis. These observations indicate that HBeAg may be one of the targets of treatment in hepatic fibrosis associated with HBV.

It is likely that extracellular matrix-producing cells other than HSCs, such as fibroblasts and myofibroblasts of the portal tract and circulating ‘fibrocytes’, also contribute to hepatic fibrosis [45]. HBV does not appear to infect all hepatocytes and thus it replicates in limited hepatocytes, leading to a heterogonous distribution of HBV proteins [46]. In chronic hepatitis B infection, the number of HSCs in asymptomatic carriers is lower than in cirrhotic patients because the number of HSCs in the space of Disse in the normal liver is less than that in the injured liver [8]. Thus, factors other than HBeAg contribute to hepatic fibrosis in chronic hepatitis B infection.

NF-κB protects activated stellate cells against apoptosis [47]. MG132 is an NF-κB suppressor and inhibits the phosphorylation of IκBα [14]. IL6 levels were 2.1 ng/mL and 1.85 ng/mL in the conditioned media from LX-2 treated with conditioned media from HepG2 or HepG2.2.15, respectively, in the absence of MG132, whereas those in the conditioned media from LX-2 treated with conditioned media from HepG2 or HepG2.2.15 cells were undetectable in the presence of MG132. Similarly, both MCP-1 and IL1β levels in the conditioned media from LX-2 cells treated with conditioned media from HepG2 or HepG2.2.15 cells were undetectable in the presence of MG132 (data not shown). Thus, these cytokines do not appear to be involved in MG132-induced HSC apoptosis. These results have prompted us to examine the AP-1 signaling pathways rather than the NF-κB signaling pathways, which are associated with the production of cytokines. We also observed no significant differences in cellular-FLICE-inhibitory protein (c-FLIP) expression between the treatment groups in the presence of MG132 (data not shown). It was reported that MG132 sensitizes tumor-necrosis factor-related apoptosis-inducing ligand (TRAIL)-resistant prostate cancer cells by activating the AP-1 signaling pathway, repressing the anti-apoptotic molecule c-FLIP(L) [17]. HBV infection blocks HSC apoptosis through the inhibition of AP-1 activation, and may evoke the activation of HSCs, contributing to the progression of hepatic fibrosis. In HSCs, the AP-1 signaling pathway could be one of the therapeutic targets in HBV-associated hepatic fibrosis. Although the exact mechanisms of the action of proteasome inhibitors are not yet fully defined, there are several pathways that appear to be important in the selectivity for malignant cells [48]. Further studies about similar pathways in HSCs will also be needed.

ER stress pathways are linked to cell death and have been implicated in several diseases [49,50]. The present study indicates that HBV infection might enhance the expression of ER stress-related genes, which are associated with cell proliferation in LX-2 cells. HSC activation in vivo is a very complicated and multifactorial process in which ER stress is surely involved [49,50]. These observations suggest that the blockade of ER stress-related pathways may also provide a new therapeutic option for HBV-related hepatic fibrosis.

Interferon-α treatment results in a decrease in the number of activated HSCs in the liver tissues of patients with chronic hepatitis B [51], and it was also reported that AP-1-dependent genes are increased in sera from HBV-infected individuals after interferon-α treatment [52]. Thus, the number of HSCs seems to be associated with AP-1-dependent genes. However, it may be difficult to confirm our observation in the present study of the lower expressions of AP-1 and c-Jun in HSCs of HBV-infected patients, as the performance of liver biopsy in healthy individuals would be out of the question due to legality issues. HBV replication could be well controlled by antiviral treatment, but conversion from established cirrhosis to non-cirrhotic liver in HBV-infected patients is still difficult. TRAIL and AP-1 signaling pathways are attractive targets, as they could play a role in HSC apoptosis [53]. These mechanistic insights could shed new light on a treatment strategy for HBV-associated hepatic fibrosis. In conclusion, HBV may contribute to the progression of hepatic fibrosis through the inhibition of HSC apoptosis. Possible mechanisms of the involvement of HBV virion produced from hepatocytes and the AP-1 signaling pathway in HSCs may also be significant determinants in the progression of hepatic fibrosis associated with HBV infection.
